# Unravelling the antimicrobial activity of peptide hydrogel systems: current and future perspectives

**DOI:** 10.1039/d1sm00839k

**Published:** 2021-08-24

**Authors:** Emily R. Cross, Sophie M. Coulter, Sreekanth Pentlavalli, Garry Laverty

**Affiliations:** Biofunctional Nanomaterials Group, School of Pharmacy, Queen's University Belfast, Medical Biology Centre 97 Lisburn Road Belfast N. Ireland BT9 7BL UK garry.laverty@qub.ac.uk

## Abstract

The use of hydrogels has garnered significant interest as biomaterial and drug delivery platforms for anti-infective applications. For decades antimicrobial peptides have been heralded as a much needed new class of antimicrobial drugs. Self-assembling peptide hydrogels with inherent antimicrobial ability have recently come to the fore. However, their fundamental antimicrobial properties, selectivity and mechanism of action are relatively undefined. This review attempts to establish a link between antimicrobial efficacy; the self-assembly process; peptide–membrane interactions and mechanical properties by studying several reported peptide systems: β-hairpin/β-loop peptides; multidomain peptides; amphiphilic surfactant-like peptides and ultrashort/low molecular weight peptides. We also explore their role in the formation of amyloid plaques and the potential for an infection etiology in diseases such as Alzheimer's. We look briefly at innovative methods of gel characterization. These may provide useful tools for future studies within this increasingly important field.

## Abstract 2

The use of hydrogels has garnered significant interest as biomaterial and drug delivery platforms for anti-infective applications.

## Graphical abstract

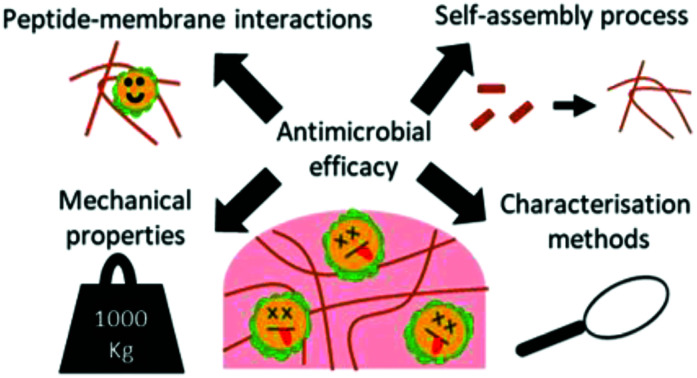

## 1. Introduction

There is a need for innovative antimicrobial platforms in order to successfully address growing concerns of antimicrobial resistance to conventional therapeutic approaches.^1^ Soft materials, especially hydrogels, are commonly employed as biomaterials for the treatment and prevention of infection. Primarily such applications include their use as wound dressings, tissue scaffolds and medical device coatings.^2^ Hydrogels are highly amenable to such use due to their similarities to the host's extracellular matrix providing: improved biocompatibility;^3^ high water content;^4^ fibrous architecture to support cell attachment and growth;^5^ sustained delivery of molecules of interest *e.g.* drugs;^6^ and a lubricious, low friction coating for increased comfort upon insertion *e.g.* urinary catheters.^7^

Antimicrobial peptides have long been studied as a potential source of new antimicrobial molecules given their role as key components of the natural innate immune response to infection.^8^ This has resulted in the clinical use of antibiotics such as colistin (polymyxin E), polymyxin B and daptomycin, primarily for topical (*e.g.* skin, lungs) rather than systemic delivery. The key advantage of antimicrobial peptides as therapeutics is their ability to inflict microbial cell death *via* multiple modes of action. The majority of peptides which demonstrate antimicrobial activity tend to be amphiphilic, composed of a variety of charged, mainly cationic, hydrophilic and hydrophobic amino acids. The bacterial cell membrane serves as their primary target *via* electrostatic and hydrophobic interactions leading to formation of ion channels, pores and leakage of cell contents. Antimicrobial peptides also act to prevent several intracellular processes including: cell division, cell wall formation; DNA, RNA, protein synthesis and metabolism; protein-folding and the action of microbial proteases.^9^ This is favourable compared to existing antimicrobial therapies which tend to exert their effect by acting upon a single microbial target or process. There is increased potential for microorganisms to overcome this single mode of antimicrobial action, thereby leading to the generation of resistant strains. The mechanism and application of antimicrobial peptides has been covered extensively by several related publications, including those from our own group,^10,11^ and therefore is not the main focus of this review.

Self-assembly has been succinctly linked to several fundamental biological processes, including the highly precise interactions of protein substrates within the enzyme active site; the ability of nucleic acids to form the helix of DNA and of lipids to form cell membranes.^12^ By definition, self-assembly is the spontaneous formation of higher ordered supramolecular structures from individual molecular units, primarily due to non-covalent intra and intermolecular interactions, for example hydrophobic, van der Waals’, π–π stacking, hydrogen bonding and electrostatic interactions.^13^ A self-assembled state is generally preferred due to higher thermodynamic stability (lower energy state) than its free less ordered form *e.g.* in solution or as disordered aggregates.^14^

**Fig. 1:**
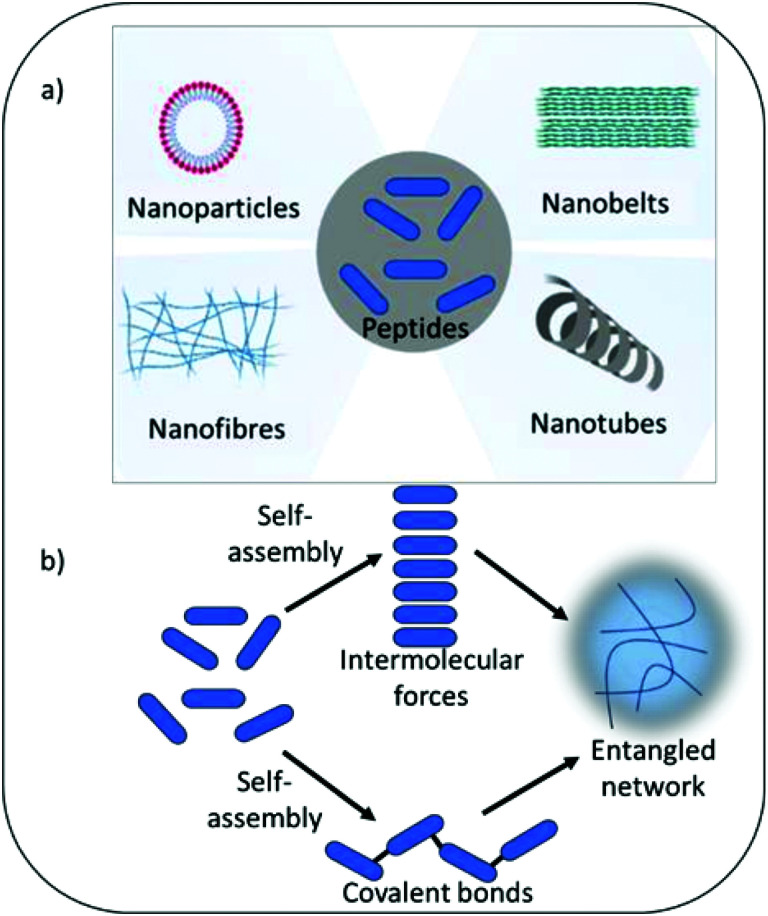
(a) Examples of the various nanostructures which may be formed during self-assembly. (b) The self-assembly process of gelation. The peptide gelators (blue ovals) self-assemble into secondary structures, commonly β-sheet and α-helical, when a trigger is applied that makes the gelators insoluble. Secondary structures can either be held together by intermolecular forces of attraction or covalent bonds. These secondary structures entangle to immobilize water which forms a gel.

Self-assembling peptide hydrogels are an emerging field of research for drug delivery,^15^ tissue engineering,^16^ cell culture and electronic applications.^17,18^ The peptide motif provides the ability to tailor assembly and hydrogel formation in response to a variety of external stimuli including pH, temperature, light, ionic strength and specific enzymes.^19^ The amphiphilic and hydrophobic nature of peptide's amino acid building blocks act as drivers for self-assembly and formation of a variety of nanostructures, including nanofibers, nanotubes, nanocages, nanobelts and nanovesicles (Fig. 1). As highlighted above, such properties are also central tenet to the antimicrobial activity of peptides. Whilst the physical process of assembly is highly defined for several peptide nanostructures, linking self-assembly succinctly to biological and pharmacological properties is an increasingly interesting area of research. This would enable the bottom-up design of next generation medical materials whereby biological, functional and pharmacological properties could be dictated by self-assembling properties in response to specific external and physiological stimuli. The development of several infections can be linked to changes in physiological properties, which could potentially be harnessed to trigger antimicrobial activity when it is most needed. For example, infection of urinary catheters commonly leads to an increase in urine pH due to the action of the enzyme urease produced by pathogenic bacteria (*Proteus mirabilis*) implicated in such infection.^20^ Urease acts on urea converting it to ammonia, thereby increasing pH within the urinary environment, leading to precipitation of urinary salts, encrustation of the catheter, blockage and resulting in device failure. In the immediate short-term (days) after catheter insertion infection can be prevented *via* inclusion of standard antimicrobials within the hydrogel coating or attached to its surface. However, after a few days the drug reservoir is often exhausted due to rapid burst release of drug from the hydrogel matrix to the surrounding environment. This results in sub-therapeutic levels of antimicrobials, with the potential for resistance to develop and loss of protection against infection. Commonly infection does not establish until 3–4 weeks after urinary catheter insertion. A hydrogel coating that was able to respond to the initial development of infection, triggered by pH change or presence of specific bacterial enzymes, should in theory provide long-term protection. Self-assembly can also enhance stability against protease degradation, reticuloendothelial system uptake and renal filtration compared to free non-assembled forms, therefore indirectly enhancing activity by prolonging exposure time and improving pharmacokinetic parameters.^21^

In the past decade, there has been increased interest in the antimicrobial activity of supramolecular peptide systems, including peptide hydrogels, with scientists attempting to succinctly link their structure to mode of antimicrobial action. Several prominent recent reviews and chapters highlight the exciting potential of using peptide hydrogels within antimicrobial applications.^22–25^ This review will delve into the existing theory surrounding peptide nanostructures with an emphasis on the different types and examples of self-assembled hydrogels and whether antimicrobial efficacy can be linked succinctly with self-assembly behaviour and mechanical properties (*e.g.* gel strength) leading to new avenues of research for biomaterial design.

## 2. Peptide–membrane interactions

**Fig. 2:**
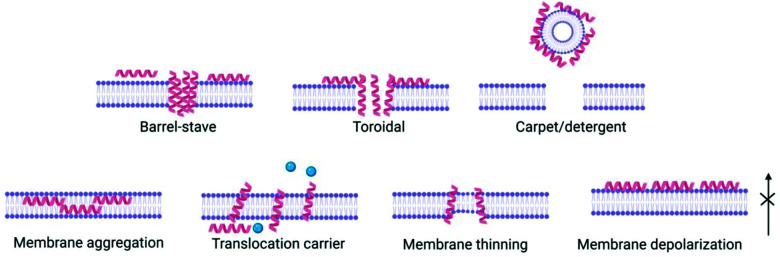
The various modes of action proposed for antimicrobial peptides' effect on bacterial cell membranes including barrel-stave, toroidal pore or carpet model, as well as less disruptive non-pore mechanisms. The mechanism of bilayer perturbation is dependent on the peptide and the lipid membrane composition, and may change depending on peptide concentration, temperature and pH.

The behaviour of peptides and proteins with biological membranes is fundamental to our underlying understanding of cell biology, development of diseases and their treatment. The biological function of proteins and peptides is inherently linked to how they assemble and the structural arrangements that they adopt.^26^ Given the microbial cell membrane is a primary target of antimicrobial peptides, there is unsurprisingly a focus on defining peptide interactions with bacterial and fungal cell membranes. The bacterial cell cytoplasmic membrane is composed of a phospholipid bilayer with a mainly hydrophobic internal and hydrophobic external face. As Le and colleagues highlight,^9^ peptide–membrane interactions can be summarized by three main phases within aqueous, physiological conditions:

(1) Amphiphilic peptides coil with hydrophobic segments shielded within an inner core. Hydrophilic electrostatic attractions are the initial focus for peptide–membrane interactions.

(2) Peptides accumulate at the membrane surface. Hydrophobic interactions drive conformational changes in the peptide structure. Hydrophobic segments of the peptide insert into the membrane bilayer.

(3) Peptides, now inserted within the membrane, interact with each other and the membrane to exert their biological effect *e.g.* pore formation, surfactant-like action (Fig. 2).

Peptide concentration, primary structure (amino acid composition/sequence) which dictates charge, hydrophobicity and secondary/tertiary structure have a direct influence on membrane interactions and therefore potential antimicrobial efficacy. Several models have been proposed for antimicrobial peptides’ impact on bacterial cell membranes: barrel stave,^27^ toroidal pore,^28^ carpet,^29^ and membrane curvature models,^30^ whereby peptide interaction results in membrane disruption above a threshold peptide to lipid concentration ratio, leading to depolarization and cell death (Fig. 2). This interaction is of great importance in order for antimicrobial peptides to exert their effect. The most common way for interactions of peptides to be studied *in vitro* are with bacterial or mammalian models of zwitterionic membranes composed of 2-oleoyl-1-palmitoyl-*sn-glycero*-3-phosphocholine (POPC), 1,2-dioleoyl-*sn-glycero*-3-phosphocholine (DOPC) and mixtures of anionic 2-oleoyl-1-palmitoyl-*sn-glycero*-3-phospho-rac-(1-glycerol) sodium salt (POPG) and 2-oleoyl-1-palmitoyl-*sn-glycero*-3-phosphoethanolamine (POPE) enabling study of peptide self-assembly and membrane interactions using techniques such as circular dichroism (CD) spectroscopy, isothermal titration calorimetry (ITC), fluorescence spectroscopy, small angle neutron scattering (SANS), small angle X-ray scattering (SAXS), rheology and nuclear magnetic resonance (NMR) (see Section 5).^31^

The secondary structure of antimicrobial peptides that form at membrane surfaces tend to fall within one of four groups: α-helical (LL-37 human cathelicidin, magainin, cecropin, pexiganan, temporins, melittin), β-sheet (α-, β-, θ-defensins), β-hairpin or loop combinations (lactoferricin B) or extended sequences with no defined secondary structure (histatins, indolicidin). The majority of antimicrobial peptides studied in aqueous solution form random coils, assembling into their secondary structure when they encounter membranes. Most α-helical peptides are amphipathic, predominantly cationic in character, enabling broad-spectrum activity *via* disruption of bacterial and fungal membranes. β-Sheet peptides tend to form more rigid, stacking arrangements, commonly stabilized by disulfide bonds, with their amphipathic character also enabling membrane disruption. Random coils lack a specific secondary structure due to a high density of specific amino acids including histidine, arginine, glycine and proline. In the presence of cell membranes, they fold to form amphipathic structures enabling disruption of membranes and/or cell entry to act on intracellular targets. Secondary structure and amphiphilicity also have a significant bearing on the ability of a peptide to self-assemble and the specific nanostructure formed, with β-sheet and α-helical and secondary structures key molecular motifs often identified. The ability of peptides to self-assemble into secondary and/or supramolecular nanostructures should therefore be related to antimicrobial efficacy. However, this ability cannot be taken as prerequisite to increased antimicrobial efficacy experimentally. Some naturally occurring antimicrobial peptides, modified structurally to include a self-assembling sequence, have demonstrated reduced antimicrobial activity upon self-assembling.^22^

For self-assembled peptide aggregates, charge density at the assembled surface and charge to surface area ratio are important additional considerations for membrane interactions,^32,33^ enabling detergent-like effect,^34^ and formation of membranous pores.^35^ For amphipathic antimicrobial peptides in aqueous environments, charged residues (*e.g.* lysine, arginine) tend to be located on the surface of the fibrous self-assembled structure, with hydrophobic residues (*e.g.* phenylalanine, tryptophan) forming the core. These localized areas of charge and hydrophobicity are important in mediating interactions at the water–membrane interface.^22,36^

An interesting concept is that hydrogel formation increases the local concentrations of drug or active moieties (*i.e.* areas of cationic charge), enabling more potent activity at bacterial cell surfaces, even if the overall drug concentration is below that of fully solubilized solutions.^37^ Initial work by Bing Xu's group studied the ability of Gram-positive selective antibiotic vancomycin to form hydrogels when conjugated to a highly aromatic pyrene group.^38^ They observed that vancomycin hydrogels were eleven times more potent than solutions of unmodified vancomycin and were effective against vancomycin resistant enterococci. It was hypothesized that vancomycin–pyrene derivatives may assemble at the bacterial cell surface even at concentrations below the experimentally observed minimum gelation concentrations. This is due to hydrogen bonding between vancomycin and bacterial cell wall l-Lys-d-Ala-d-Ala peptide motif of peptidoglycan increasing localized concentrations of vancomycin at the bacterial cell surface. Research by Ren and colleagues took this concept and the high affinity of vancomycin for l-Lys-d-Ala-d-Ala further by adding a highly fluorescent 4-nitro-2,1,3-benzoxadiazole (NBD) motif to vancomycin *via* a peptide linker.^39^ This allowed the possibility for simultaneous bacterial detection and inhibition. Vancomycin's specificity for bacterial peptidoglycan is particularly advantageous for increased targeting of bacterial (Gram-positive) relative to mammalian cells, although the susceptibility to resistance is increased. Whilst pockets of cationic charge with antimicrobial peptides demonstrated increased electrostatic attraction for anionic components of bacterial cell membranes, their affinity does appear to be less specific and it is more difficult to control the likelihood of mammalian cell toxicity.

These materials can be finely tuned so that their physical and chemical properties achieve the best peptide–membrane interaction.

## 3. Linking self-assembly, mechanical and antimicrobial properties of peptide hydrogels

There is increased interest in combining the anti-infective properties of antimicrobial peptides with the flexibility offered by natural and unnatural amino acids in order to design materials whereby molecular structure can be tailored to specific functional and pharmacological properties. Supramolecular hydrogels formed from peptide nanofiber structures offer the most promise within engineering, biomaterial and pharmaceutical applications given the use of synthetic hydrogels as coatings for medical implants,^7^ wound healing dressings,^40^ and within drug delivery.^6,41^ Over the past decade peptide hydrogels have demonstrated efficacy against a broad range of Gram-positive (*Staphylococcus aureus* including methicillin resistant [MRSA] and sensitive strains, *Staphylococcus epidermidis*) and Gram-negative bacteria (*Escherichia coli*, *Pseudomonas aeruginosa*, *Klebsiella pneumoniae*) implicated within healthcare infections and the increasing prevalence of bacteria resistant to standardly prescribed antibiotics. Although studied less frequently, their benefit within antifungal applications has also been postulated.^42,43^

It is also important to look closely at peptide nanostructures to establish a link between primary and secondary structure; assembly; mechanical and antimicrobial properties. There appears to be different structure–activity relationships and molecular mechanisms that determine antimicrobial activity for fully solubilized and nanofibrous peptides. Increasing evidence suggests that tailoring molecular conformation, assembled state and mechanical properties are key to determining antimicrobial efficacy in such systems. This review will look at existing groups of antimicrobial peptide hydrogelators to establish whether a significant link between these characteristics exists. The chemical versatility of the peptide motif provides the optimum template to modify such characteristics at a molecular level in order to design more effective and responsive antimicrobial materials.

### 3.1. β-Hairpin/β-loop systems

**Fig. 3:**
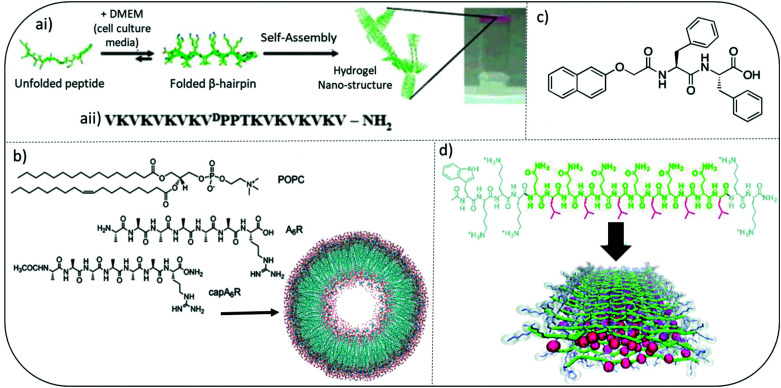
Examples of peptide hydrogel systems (ai) mechanism of folding and self-assembly for MAX1 β-hairpin hydrogel formation. Resulting gels are self-supporting as image at far right shows. (aii) Sequence of MAX1 β-hairpin. Adapted with permission from ref. 42. Copyright (2007) American Chemical Society. (b) Chemical Structures of the lipids and surfactant like peptides used to form the vesicle shown. Adapted with permission from ref. 48. Copyright (2018) American Chemical Society. (c) Chemical structure of an ultrashort peptide composed of a naphthalene (Nap) protecting group and a diphenylalanine (FF) peptide chain. (d) Colour-coded chemical structure of a multidomain peptide that can be designed to contain variable numbers of lysine residues and (QL) repeating units for the investigation of both molecular and supramolecular structure-dependent cytotoxicity and antimicrobial activity. Red: leucine; green: glutamine; grey: lysine. Adapted from with permission from ref. 45, The Royal Society of Chemistry.

Pioneering work by Schneider and Pochan initiated significant interest in developing self-assembled peptide hydrogels with inherent antibacterial efficacy. They introduce MAX and MARG twenty residue peptides where MAX1 has a hydrophilic face composed exclusively of lysine side chains whereas, MARG1 has a mixture of lysine and arginine residues. These salt-responsive β-hairpin forming peptide hydrogels demonstrated broad spectrum activity against a range of clinically relevant pathogens, including staphylococci*, E*. *coli* and *P*. *aeruginosa* (Fig. 3a).^44,45^ Their design consists of two eight-residue peptide sequences of alternating valine and lysine amino acids connected by a tetrapeptide motif (VpPT) that allowed a type II′ β-turn to form in cell media containing salts (∼160 mM), thereby creating an amphiphilic β-hairpin with hydrophobic and opposing charged faces when fully assembled. Interestingly, when mammalian NIH3T3 fibroblasts were cocultured with a mix of *Stenotrophomonas maltophilia* and *Achromobacter xylosoxidans* bacteria, MAX1 hydrogel was able to selectively inhibit bacterial growth but allow mammalian cell adhesion and proliferation.^44^

Mechanical properties, antibacterial efficacy and mammalian cell toxicity were altered by changing the type and number of constituent cationic amino acid residues. For example, MAX groups containing only lysine residues and MARG containing both arginine and lysine residues. To denote the increase in arginine residues Schneider's group introduce the term PEPnR where *n* is the number of arginine residues within the twenty residue peptide (*e.g.* PEP8R refers to a peptide chain containing 8 arginine and 12 lysine residues).^46^ These cationic motifs provided the ability for bacterial membrane disruption *via* a mechanism termed “contact killing.” Further characterization with the PEP variants produced some interesting properties with PEP8R representing greater numbers of lysine to arginine substitutions studied. It was found that PEP6R (VKVRVRVRVpPTRVRVRVKV) formed stronger gels than either PEP8R, PEP4R or PEP2R. The group concluded that in order to achieve broad spectrum antibacterial activity (Gram-positive and Gram-negative) a minimum of four arginine (*i.e.* PEP4R) residues were required and that antibacterial activity improved relative to increased concentration of peptide.^46^ Gel strength was not attributed directly to improved antibacterial efficacy in this case. PEP2R only had significant efficacy against *S*. *aureus*. There was little difference in the antibacterial action of PEP8R, PEP6R and PEP4R but PEP8R did express increased mammalian cell toxicity, attributed to increased presence of cationic amino acid arginine. This was likely providing reduced selectively for anionic bacterial membranes relative to neutrally charged mammalian forms.

In a related study, the Zhao group studied a peptide composed of two cationic antibacterial peptide sequences, (KIGAKI)_3_-NH_2_, covalently linked by a central tetrapeptide YpPG β-turn loop.^47^ This enabled the formation of a β-sheet forming hydrogel with activity against Gram-negative *E*. *coli*. Similar to Schneider and Pochan's group of cationic β-hairpin peptides, the researchers hypothesized antibacterial activity to be due to “contact killing” due to cationic (KIGAKI)_3_-NH_2_ peptide's presence at the external hydrogel surface.

In future, a deeper understanding of peptide–membrane interactions, fundamental to cell biology, the development of infectious diseases and their treatment, will enable a peptide catalogue to be created that is tailored to specific species and strains of bacteria implicated within individual infections.

### 3.2. Multidomain peptides

A variety of multidomain peptides (MDP) have been explored, combining self-assembly and antimicrobial activity *via* a central amphiphilic portion (B), flanked by two charged termini (A), forming an ABA peptide sequence. The Dong group studied the antibacterial properties of MDP-1 (WK_2_(QL)_6_K_2_), MDP-2 (WK_3_(QL)_6_K_2_), and MDP-3 (K_3_W(QL)_6_K_2_) (Fig. 3d).^48^ MDP-2 (WK_3_(QL)_6_K_2_) demonstrated the most potent antibacterial activity against planktonic forms of Gram-positive *S*. *epidermidis* and *S*. *aureus* and Gram-negative *E*. *coli* and *P*. *aeruginosa*. Interestingly despite having the same overall charge density as MDP-2, MDP-3 demonstrated reduced activity. The authors suggested charge density had minimal impact on antibacterial activity when the peptide existed in its soluble form and antibacterial potency was also inversely related the ability of MDP's to self-assemble into β-sheets. Enhanced supramolecular order, for example partial β-sheet formation, appeared to compromise the antimicrobial activity of the soluble MDP nanofibers. This tends to oppose some more recent findings for fully assembled forms and it is likely that the exact location of charge density within these nanofibers may have a more direct influence than overall charge. Further and more recent work on assembled MDP confirmed a difference in activity for assembled hydrogels compared to soluble forms.^49^ More densely packed nanofibrous structures were observed for MDP variants K_2_W(QL)_6_K_2_ and WK_2_(QL)_6_K_2_ and these enabled greater trapping of bacteria within the peptide gel matrix due to their higher mechanical strength observed through measurement of their rheological storage modulus. Reduced bacterial motility was due to enhanced suctioning force within these nanofibrous hydrogels and greater interaction between bacterial cell membranes and cationic lysine present on the MDPs. In a follow-on study by the Dong group they were able to demonstrate self-assembly into β-sheet nanofibers was crucial in conferring antibacterial activity and also selectivity (*vs.* mammalian cells) in hydrogels, including a modified MDP containing D enantiomeric (for W and K) and l-amino acids, called D-W362 (wk_3_(QL)_6_k_2_).^50^ Linking distinct self-assembled architectures, such as β-sheet nanofiber formation, to cell selectivity is a key area of research to inform future design of antimicrobial peptide-based materials.

### 3.3. Amphiphilic surfactant-like peptides

Amphipathic surfactant-like peptide have also been studied as antibacterial hydrogels. As observed with MDPs, I_3_QGK, A_6_K, A_9_K, A_9_K_2_, V_6_K, V_6_K_2_, I_3_K and L_3_K rely on the cationic amino acid lysine to provide electrostatic interactions with bacterial cell membranes, with the presence of neighbouring hydrophobic amino acids enabling surfactant-like disruption of membranes and hydrogel forming capability.

Early studies by Chen and colleagues using amphiphilic surfactant-like peptides A_3_K, A_6_K, and A_9_K suggested a clear link between peptide self-assembly, their ability to penetrate membranes and antibacterial efficacy (Fig. 3b).^51^ The hydrophobic tail length, provided for by alanine, was particularly important. Castelletto and Hamley characterized a surfactant-like peptide variant whereby lysine was replaced by arginine to form A_6_K, including terminal alanine capped CH_3_CONH-capped modifications,^52,53^ and A_9_R.^54^ The capped A_6_K variant, CH_3_CONH-A_6_R, formed nanofibrils, whilst the uncapped A_6_R assembled into nanotapes. Using model membranes CH_3_CONH-A_6_R was able to selectively interact with the anionic lipid [2-oleoyl-1-palmitoyl-*sn-glycero*-3-phospho-rac-(1-glycerol)] (POPG) in POPG/POPE [POPE: 2-oleoyl-1-palmitoyl-*sn-glycero*-3-phosphoethanolamine] vesicles.^53^ CH_3_CONH-A_6_R also showed selective antibacterial activity against Gram-positive food-borne pathogen *Listeria monocytogenes*, compared to mammalian fibroblasts. Uncapped A_6_R showed broader antibacterial activity against *S*. *aureus, L*. *monocytogenes*, and *E*. *coli*. Interestingly CH_3_CONH-A_6_R was able to form β-sheets in the presence of anionic lipid membranes whereas A_6_R was not. This research highlights how small changes (CH_3_CONH-A_6_R *vs.* A_6_R) in charge density and distribution can ultimately impact a peptide's interaction with lipid membranes bearing variable charge and therefore antimicrobial activity. A_9_R was able to self-assemble into hydrogels and possessed greater antibacterial activity against Gram-negative *P*. *aeruginosa* than Gram-positive bacteria. Both of these properties were linked to the ability of A_9_R to form β-sheet fibers.^54^ Membrane thinning was observed through the use of model membranes without membrane lysis. Intriguingly they also demonstrated that A_9_R was able to stabilize oil in water (o/w) emulsions by forming a β-sheet fibril coating. This ability was reversed upon exposure to protease enzyme elastase. The authors hypothesized the potential use of A_9_R within protease-responsive antimicrobial peptide emulsions, whereby the de-emulsification upon protease exposure would lead to triggered release of antimicrobial peptide.

The Kumar group's work on cationic amphiphilic self-assembled peptide (CASP) hydrogels characterized bacterial membrane interactions with peptides nanofibers using a combination of microsecond-time scale coarse-grained simulations and peak force quantitative nanomechanical atomic force microscopy.^55^ The peptide nanofibers of CASPs were shown to stiffen, contract and buckle *P*. *aeruginosa* bacterial membranes upon contact. The resulting membrane disruption significantly impacted the osmotic balance between the intracellular and extracellular milieu leading to cell lysis. More recently, the Kumar group characterized a series of variants of low molecular weight FF peptides, namely anthranilamide-based peptide hydrogels.^56^ Interestingly they studied the relationship of gel stiffness, as observed by rheology and antibacterial efficacy, using *S*. *aureus* as a model pathogen. Higher gel strength, most notably through higher *G*′ values provide mechanical support to individual fibers/fibrous networks to direct specific chemical functional groups, *e.g.* cationic charge, against restricted bacterial cells, providing improved bactericidal activity as similarly demonstrated for MDPs.^49^

### 3.4. Ultrashort, low molecular weight peptides

Ultrashort peptides, defined as peptide or peptidomimetic molecules containing seven or less amino acid monomer units, have been of particular interest given the potential to provide clinical anti-infective efficacy alongside improved ease of synthesis and reduced cost of manufacture.^57^ These are key factors in improving ability to translate these formulations to patients. The use of groups with high aromaticity, most notably phenylalanine and a neighbouring naphthalene (Nap) or 9-fluorenylmethoxycarbonyl (Fmoc), is central to their ability to form self-assembled supramolecular hydrogels (Fig. 3c). Given its use as a protecting group within standard solid-phase peptide synthesis, Fmoc peptides were the focus for initial study of short dipeptide gelators. Its inclusion enabled a reduction in synthetic steps and therefore potential for improved synthetic yield and purity. Interest in antimicrobial hydrogels within for these molecules stemmed from a combination of Schneider's β-hairpin antimicrobial peptide hydrogel work and research from Reches and Gazit. They observed diphenylalanine (FF) to be a minimum motif for formation of nanostructures, in this case nanotubes, and was the central motif for β-amyloid polypeptide formation in diseases such as Alzheimer's.^44,58^ The FF motif self-assembles to form peptide-based nanotubes but does not have sufficient intermolecular interaction alone to form hydrogels. FF nanotubes have recently demonstrated efficacy against both free flowing planktonic and surface attached biofilm forms of bacteria implicated in hospital infections.^59,60^ Membrane disruption and up-regulation of bacterial stress-response regulons have been hypothesized to be the main mechanism of action of FF nanotubes. Phenylalanine's aromatic group provides for increased hydrophobic character within the primary peptide sequence. Amide groups or the inclusion of specific charged amino acids, most commonly of cationic/basic character, enable a hydrophobic: charge balance to be achieved. This balance is hypothesized to be important to exerting antimicrobial selectivity and effect, most notably driving initial electrostatic interaction with anionic bacterial membranes and is best demonstrated when correlating antimicrobial activity to acyl chain length for ultrashort lipopeptides.^10^ A sequential increase in acyl chain length, *via* covalent attachment of a variety of fatty acid motifs to the amino terminus of a short peptide motif, has also yielded a promising group of antimicrobial lipopeptides with hydrogel forming capability and amphiphilic character.^61,62^ The Das group synthesized a variety of promising low molecular weight l-tryptophan monopeptides of varying hydrocarbon chain lengths (C_10_–C_18_).^62^ Further work by the group led to the wider study of dipeptides containing l-proline, l-tryptophan and l-phenylalanine with C_14_ tails.^63^ They demonstrated potent (μg mL^−1^) efficacy against a variety of pathogenic bacteria and fungi, alongside an ability to form gels in water. Although antimicrobial activity was not specifically tested for gel forms. The use of highly aromatic sequences has commonly been employed to create self-assembled antibacterial hydrogels from low molecular weight motifs. Das and colleagues also studied the antimicrobial characteristics of LF dipeptides capped with 9-anthracenemethoxycarbonyl (Amoc).^64^ Interestingly these also possessed additional anti-inflammatory activity. This would be important to improve biocompatibility within the host's environment upon administration as a biomaterial implant. Linking mechanical properties of these gels, for example using rheological methods, to biological characteristics would provide useful insight as to their potential for future use biomedical applications.

Baral and colleagues performed similar assessment to compare the antibacterial activity of two low molecular weight dipeptides containing l-phenylglycine or l-phenylalanine sequences.^65^ One of these was able to form hydrogels in phosphate buffer (pH 6.0–8.8) and the other was not. Hydrogel-forming ability was endowed by a single switch from l-phenylglycine to l-phenylalanine and was accompanied by a significant increase in bactericidal character specifically against Gram-negative bacteria (*P*. *aeruginosa* and *E*. *coli*). This single amino acid substitution suggested there was a potential link between hydrogel forming ability and antibacterial potency, however the exact nature of this relationship, for example in terms of molecular arrangement when exposed to bacterial cells, needs to be explored further.

Reches developed the diphenylalanine motif further by studying the antibacterial and self-assembly characteristics of a series of fluorinated phenylalanine analogues which provided for antifouling properties (prevention of protein and bacterial adherence) similar to the anti-adhesive properties of Teflon.^66^ Ensuring sufficiently stable attachment to implant materials, whilst maintaining antifouling properties, is a major challenge in the development and use of peptide hydrogels within the field of medical device coatings. Reches incorporated the main amino acid motif of mussel adhesive protein, 3,4-dihydroxy-l-phenylalanine (DOPA) to improve adherence to various polymeric surfaces. Hydrogels of Fmoc-pentafluoro-phenylalanine (Fmoc-F_5_-F) were recently shown to be of use in dental restorative applications.^67^ When combined with a resin-based composite, Fmoc-F_5_-F provided the ability to enhance dental remineralization with antibacterial efficacy also demonstrated against the periodontal pathogen *Streptococcus mutans*. This research highlights the potential of fluorinated ultrashort peptide hydrogels within the biomaterial and dental fields. Further work is required to study their efficacy in preventing longer-term infection development, for example weeks after administration.

Studies by our own group have shown that the addition of two basic lysine residues to Fmoc-FF-OH and Nap-FF-OH was sufficient to create a peptide hydrogel with broad spectrum antimicrobial activity and efficacy against established biofilm forms of bacteria implicated in a wide range of medical device infections.^68,69^ Whilst these molecules demonstrate promise for clinical healthcare applications, toxicity at hydrogel forming concentrations (≥0.5% w/v) remains a key consideration for any future use, especially for treatment and/or prevention of systemic infections. A promising preliminary toxicity profile was established for Nap-FFKK-OH using a simplified *in vivo* waxworm model.^70^ More recently a NapFFεKεK-OH variant of NapFFKK-OH, where the epsilon (*ε*) amino group forms part of the peptide bond rather than the standard amino grouping, demonstrated enhanced cell biocompatibility with dental pulp stem/stromal cells enabling production of an angiogenic secretome.^71^ Despite a comparative reduction in activity against relevant bacterial biofilms, these hydrogels demonstrate promise as materials within tissue engineering, stem cell delivery and cell-based pulp regeneration due to improved cell compatibility.

Previous to our own and the work of other groups within ultrashort antimicrobial peptide gelators, Bing Xu's group studied the antibacterial action of phosphorylated Nap-FFY(PO_3_H_2_)-OH.^72^ This solubilized peptide was shown to diffuse across the cell membrane of *E*. *coli* and was dephosphorylated intracellularly by phosphatase enzymes present within the bacterial cytoplasm. Dephosphorylated Nap-FFY-OH self-assembled intracellularly due to enzyme-triggered gelation. Interestingly this enabled inhibition of bacteria by increasing the viscosity of the cytoplasm and potentially blocking intracellular biomolecular pathways. This was the first suggestion that increased viscosity may have an impact on antibacterial efficacy, albeit in an intracellular context and provided a new mode of action for antimicrobial gelators. The Ulijn group followed on from this work to demonstrate similar efficacy against *E*. *coli* for a variety of phosphorylated Fmoc-protected dipeptide amphiphiles (FY, YT, YS, YN, YQ).^73^ These dipeptides were also phosphatase responsive hydrogelators. The most hydrophobic motif, Fmoc-FY-OH, was shown to accumulate and self-assemble intracellularly. Fmoc-YT-OH, Fmoc-YS-OH, Fmoc-YN-OH and Fmoc-YQ-OH being more hydrophilic in character, accumulated extracellularly outside the bacterial cell. Despite the difference in physicochemical properties and solubility, antibacterial activity was not significantly different among peptide sequences studied. This group of peptides hold significant promise for the future development of antibacterial peptide hydrogelators responsive to specific bacterial enzymes. This approach to application-based research was harnessed by the Boulmedais group who developed the phosphorylated Fmoc-tripeptide, FmocFFY(PO_3_H_2_) to self-assemble in response to alkaline phosphatase functionalized silica nanoparticles deposited on a model medical device surface. Interestingly assembled Fmoc-FFY demonstrated greater antibacterial activity than free phosphorylated Fmoc-FFY(PO_3_H_2_) solutions, once again suggesting a link between assembly and antibacterial efficacy.^74^

More recently Chauhan and Singh characterized a series of Fmoc-ff-CONH_2_ gelators containing arginine, Fmoc-Rff-CONH_2_ (RFF), and histidine, Fmoc-Hff-CONH_2_ (HFF).^75^ Each were formulated into gels using DMSO rather than pH-triggered methods, primarily due to the presence of an amide rather than a carboxylic acid terminus. This negated the ability to dissolve peptide at high pH and formulate hydrogels slowing by reducing pH to below p*K*_a_.^76^ Fmoc-Rff-CONH_2_ demonstrated the most effective broad spectrum activity against planktonic forms of *E*. *coli* and *S*. *aureus* for up to 72 hours. The use of d-phenylalanine residues provided for enhanced proteolytic stability, an important property for potential *in vivo* use and an increasingly important property to assess. There is also the scope to include peptidomimetic or peptide-like units within the peptide primary structure in order to endow resistance to host and microbial proteases. The Sarojini group studied a series of ultrashort cationic tri- and pentapeptides sequences inspired by the lipopeptide battacin.^77^ These peptidomimetic variants incorporated combinations of aromatic Fmoc- or acylated cationic d-diaminobutyric acid and lysine variants conjugated to naphthylalanine. Fmoc-d-Dab-Dab-1-Nal-NH_2_ (HG2.81) and diphenylacetyl-d-Dab-Dab-1-Nal-NH_2_ (HG2.75) tripeptides showed greatest promise as antibacterial hydrogel forming peptides with low (1.56–6.25 μM) MICs against *S*. *aureus* and *P*. *aeruginosa*. Both peptides demonstrated significant cell cytotoxicity against human dermal fibroblasts above 250 μM, highlighting the difficulty in balancing antimicrobial efficacy with potential toxicity, particularly in the higher concentrations required for systemic administration. Similarly, the Gupta group included unnatural β-alanine residues, alongside the use of a natural l-α form of lysine, to improve proteolytic stability.^78^ The pentapeptide Lys-βAla-βAla-Lys-βAla demonstrated broad spectrum antibacterial efficacy due to its amphiphilic nature, alongside an ability to self-assemble into nanovesicles. However, hydrogel formation was not observed.

Research in ultrashort peptide hydrogelators has driven interest in finding a minimum motif for both hydrogel formation and antimicrobial activity. Single Fmoc-protected amino acids, Fmoc-F and Fmoc-L, have also demonstrated selective antibacterial activity against Gram-positive pathogens *Bacillus subtilis* and *S*. *aureus* when mixed to form hydrogel co-assemblies.^79^ No activity was demonstrated for Gram-negative bacteria *P*. *aeruginosa* and *E*. *coli*. Cell wall and membrane disruption was again thought to be the main mode of action for these peptides. Fmoc-L was hypothesized to provide antibacterial activity, due to increased ability of leucine to interact with Gram-positive staphylococcal membranes *via* a non-electrostatic interaction, although no specific binding studies were performed to confirm this. Fmoc-F was mainly attributed to driving hydrogel formation *via* π–π intermolecular stacking and enabling the sustained release of Fmoc-L. The presence of the Fmoc group enhanced leucine's antibacterial efficacy by providing increased hydrophobicity, compared to unprotected leucine. Interestingly Fmoc-F gels alone did not show significant antibacterial activity. Fmoc-L did demonstrate moderate inhibition but less than Fmoc-F/Fmoc-L co-assemblies. However, a more recent study by Gahane and colleagues has demonstrated antibacterial efficacy for Fmoc-F hydrogels alone against *S*. *aureus* strains, including methicillin resistant strains.^80^ They postulated that the antibacterial activity of Fmoc-F is due to the release of solubilized Fmoc-F fragments from the hydrogel. Fmoc-F demonstrated antibacterial effects that were linearly correlated with its surfactant properties, for example its critical micelle concentration was deemed similar to its value for minimum bactericidal concentration (MBC). At higher hydrogel/micelle forming concentrations, Fmoc-F was shown to trigger osmotic and oxidative stress in Gram-positive bacteria in order to alter membrane integrity, similar to that recently observed with self-assembled FF nanotubes.^59^ In more solubilized forms, at concentrations below critical micelle concentration, Fmoc-F demonstrated inhibition of Gram-positive bacterial growth *via* cell entry and reduction of glutathione levels.

### 3.5. Combining antimicrobial peptides with self-assembling peptide sequences

An increasingly common strategy is to combine naturally occurring antimicrobial peptides with a self-assembling moiety. RADA16, derived from alternating sequence of cationic arginine (R), hydrophobic alanine (A) and anionic aspartic acid (D), is a self-assembling peptide hydrogel utilized for a variety of biomedical applications, including tissue repair and haemostatsis.^81^ Huang and colleagues combined the self-assembling property of RADA16 with the short antimicrobial peptide Tet213 (KRWWKWWRRC).^82^ Tet213 was sufficiently low molecular weight so as to not negatively impede RADA16's ability to self-assemble, whilst providing additional antibacterial activity against *S*. *aureus*. The authors therefore hypothesized a role for this formulation in osteomyelitis. They also observed that Tet312 was released from RADA16 in a sustained manner in order to provide antibacterial protection for an increased duration. It is unclear whether self-assembly or the presence of nanofibers provided an additional benefit but this would warrant further investigation. Lombardi and colleagues utilized a similar approach to enable WMR (WGIRRILKYGKRS-NH_2_), a modified variant of the marine-derived antimicrobial peptide myxinidin, to self-assemble into an amphiphilic nanofibrous architecture with activity against Gram-negative bacterial (*P*. *aeruginosa*) and fungal (*Candida albicans*) biofilms.^83^ The addition of an aliphatic alanine sequence with a lipid tail (C_19_H_38_O_2_) enabled self-assembly. This hydrophobic sequence was situated within the fiber interior, with the external fiber surface decorated with the antimicrobial WMR motif. It was unclear as to whether intermolecular interactions were sufficient to allow hydrogel formation at concentrations studied or whether the amphiphilic fibrous structure provided additional ability to “entrap” microbial cells. More recently a simplified approached endowed hydrogel forming ability alongside broad-spectrum antibacterial activity by conjugating palmitic acid to the amphiphilic nonapeptide (NH_2_-NAVSIQKKK-CONH_2_). This hydrogel was able to disrupt cell membranes, including the outer membrane of Gram-negative bacteria, resulting in cell death.^61^

The chemical versatility of peptides also enables the incorporation of specific motifs to enhance binding to biological molecules of interest, for example the common use of cell recognition RGD motif to improve cell attachment and growth.^84^ In an antibacterial context, the Webster group formed self-assembling nanorods containing heparin-binding Cardin–Weintraub motif peptide (AKKARK)_2_ attached to a C_16_-V_4_K_4_G lipopeptide sequence.^85^ This sequence was hypothesized to improve initial attachment of the lipopeptide with anionic bacterial cell membranes. Whilst hydrogel formation was not observed, this sequence was able to assemble into nanorods composed of β-sheet secondary structures. Of particular note this peptide demonstrated antibacterial activity against Gram-positive methicillin resistance *S*. *aureus* (MRSA) in both non-assembled and assembled forms. However, Gram-negative *E*. *coli* only showed activity in the nanorod form at concentrations above the critical micelle concentration (45 μM), evidence that self-assembly was a requirement for bactericidal action in Gram-negatives. They were also able to link improved binding to lipopolysaccharide, a key component of the Gram-negative outer membrane, with self-assembly. Using TEM imaging, local membrane disruption was demonstrated by using nanorods against Gram-positive bacteria and blisters/pores on membranes of Gram-negative bacteria, similar to our own observations with FF nanotubes and staphylococcal, *E*. *coli* biofilms.^60^

Recently Thota and colleagues demonstrated that the efficacy of a weak cationic antimicrobial peptide (IN4) could be improved by attaching it to a fibril-forming α-helical coiled-coil peptide (FF03).^86^ Hydrogel formation was not observed but the fibrous scaffold enhanced the presentation of cationic peptide to bacterial membranes with improved efficacy relative to the unconjugated AMP analogues and reduced toxicity to mammalian cells. The cationic charge density was more readily accessible to interact with anionic bacterial cell membranes *via* stronger electrostatic interactions. In similar work, Beter *et al.* observed that bacteria accumulate more rapidly on peptide nanofibers relative to free soluble peptide.^87^ Improved antibacterial activity may also be due to an increasing volume of interactions between cationic peptide residues and increased bacterial numbers.

### 3.6. Peptide hydrogels as delivery vehicles for standard antimicrobials

Ultrashort peptide hydrogels have also been employed as a delivery matrix for standard antimicrobials. The Marchesan group utilized a 0.2% w/v d-leucine containing tripeptide (lFF) to deliver the antibiotic ciprofloxacin. Interestingly peptide gel controls (no ciprofloxacin) also demonstrated mild antibacterial effect against the Gram-negative isolates studied (*E. coli* and *Klebsiella pneumoniae*). Antibacterial efficacy was further improved when ciprofloxacin was physically encapsulated at concentrations of 30% w/w within the lFF gel.^88^ It may be possible that the peptide gel acts synergistically with ciprofloxacin to improve activity against Gram-negative bacteria.

Silver ions (Ag^+^), adhered to peptide nanofibers by electrostatic attraction, were similarly utilized alongside Fmoc-FFECG peptide gels. This combination provided broad-spectrum antibacterial activity against both Gram-positive (*Bacillus subtilis*) and Gram-negative bacteria (*E*. *coli*).^89^ However, no peptide-only control was tested to establish if antibacterial efficacy was demonstrated by Fmoc-FFECG gels alone. This would provide an interesting follow-on study, given more recent developments in the field, to study if synergistic activity with Ag^+^ was observed. This may be possible given that single Fmoc-protected amino acids (Fmoc-L, Fmoc-P, Fmoc-A, Fmoc-H), complexed with Ag^+^ to create metallohydrogels, were recently observed to possess potent activity against both Gram-positive (*S*. *aureus*) and Gram-negative (*E*. *coli*) bacteria in both cell and mice models.^90^ The Fmoc amino acids and Ag^+^ acted as a synergistic combination for antibacterial action by destabilizing bacterial cell membranes but also Ag^+^ enhanced the formation and stability of Fmoc amino acid hydrogels. Interestingly Fmoc-H combined with Ag^+^ was the only metallohydrogel to demonstrate reduced efficacy compared to Ag^+^ alone, which the researchers stated was due to the relatively weak mechanical strength of the Fmoc-H + Ag^+^ gel.

The promising versatility of self-assembled peptide hydrogels as combined inherent antimicrobial platforms and drug delivery vehicles is highlighted by the work of Koch *et al.*^91^ They studied two peptide hydrogels, an anionic QQRFEWEFEQQ sequence (P11-4) and a cationic ornithine (O) motif containing OQOFOWOFOQO (P11-28/29) for use in dental regenerative applications. The emphasis was on characterizing the peptide hydrogels’: (i) antibacterial activity against the periodontal pathogens, Gram-positive *Streptococcus sanguinis* and Gram-negative *Porphyromonas gingivalis*; (ii) ability to act as a regenerative scaffold for human dental follicle stem cells and (iii) drug delivery potential using a set of common dental antimicrobial agents (chlorhexidine, doxycycline, tetracycline). Mainly attributed to its anionic character, P11-4 demonstrated no antibacterial activity alongside a good biocompatibility profile with dental stem cells (good osteogenic differentiation and cell growth). Whilst P11-28/29 demonstrated broad-spectrum antibacterial activity it provided only intermediate regeneration capacity (low cellular growth rates but good osteogenic differentiation), likely due to its high cationic character. Both P11-4 and P11-28/29 provided sustained antimicrobial drug delivery due to the presence of fibrous hydrogel networks. The improved regenerative potential of P11-4 deemed it a more suitable choice for further investigation with the ability for antibacterial efficacy deemed less important as it could be tailored using one or more of the conventional antibiotics studied.

Hydrogen sulphide (H_2_S) has been used to treat wounds and inflammation by promoting angiogenesis however, its potential antimicrobial effect is less well researched. Matson and co-workers created a H_2_S-releasing dipeptide hydrogel. S-FE self-assembled into a hydrogel composed of nanoribbons driven by both β-sheet formation and π–π stacking interactions. This hydrogel showed antimicrobial effects in *in vitro* assays with *S. aureus*, eliminating 97% of bacterial burden.^92^ In comparison to the non-sulphur releasing control this hydrogel had a greater bactericidal efficacy. This is another example of peptide hydrogels displaying both antimicrobial properties from its inherent structure and as a delivery vehicle for antimicrobial agents. The combination of hydrogels displaying inherent antimicrobial structure and acting as delivery vehicles will provide promising new perspectives to treat wound infections.

## 4. The potential link between amyloid peptides and antimicrobial peptides

There has been significant interest in the self-assembly of amyloid peptides and the subsequent formation and cerebral deposition of amyloid plaques. Deposition of such plaques results in several neurodegenerative disorders and diseases such as Alzheimer's, Parkinson's, Creutzfeldt–Jakob and Huntington's diseases as well as fronto-temporal dementia.^93–95^ Amyloid peptides form fibrillar nano-structures which self-assemble under specific environmental stimuli such as changes in temperature, pH and ionic strength.^96^ The resulting structures often differ substantially from the native and functional fold of the secondary, tertiary and quaternary proteins. These fibrillar structures present clinically as an accumulation of extended insoluble fibrillar deposits within cerebral tissues.^93^ The fibrils are characterized by the cross β-sheet motif with the conserved FF dipeptide motif responsible for driving organisation into higher ordered states.^97^ The exact mechanism by which amyloid peptides cause disease remains elusive.^93^

Increasing evidence for a link between microbial infection and the various diseases mentioned earlier in this section has led to interest in determining whether a specific link exists between antimicrobial peptide self-assembly and amyloid plaques.^98^ Given the importance of this link and the potential clinical implications we believe there will be a significant increase in research within this area in the near future and as such we have given amyloid peptides its own section. Research to date has primarily focused on identifying similarities and differences between the mechanism of action of antimicrobial peptides and amylogenic peptides.^99,100^ Such research includes how antimicrobial peptides could be harnessed to interfere with the aggregation of amyloid fibers and how antimicrobial peptides could be designed to suppress the formation of these fibers and the resultant neurotoxic effects.^99,100^ Interestingly, amphipathic antimicrobial peptides and short amyloid-related peptides have been shown to share a similar high frequency of aromatic amino acids.^59^ This has been implicated in both the acceleration and stabilization of amyloidogenic assemblies.^101–103^ Moreover, amyloid peptides and proteins including serum amyloid A and β-amyloid have been shown to display antimicrobial activity *in vitro* and *ex vivo*.^104,105^

**Fig. 4:**
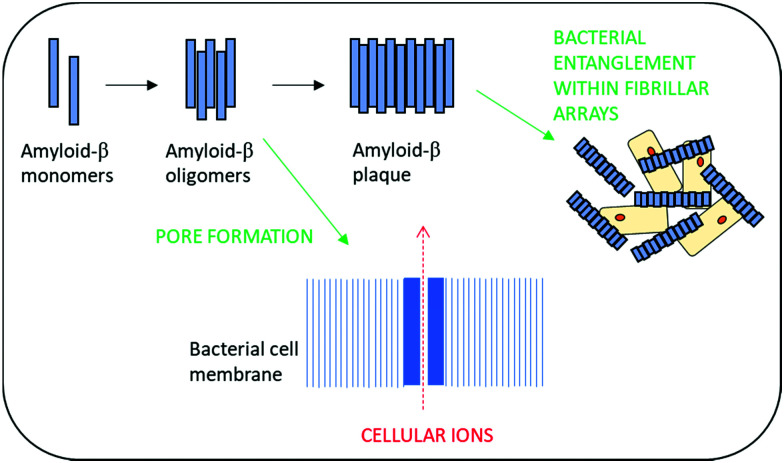
The suggested modes of antimicrobial action of amyloid-β peptides.

Amyloid-β peptides have recently been shown to exhibit broad spectrum antimicrobial activity against Gram-positive and Gram-negative microorganisms as well as activity against fungal pathogens.^105,106^ This has led to the hypothesis that the development of amyloid plaques within neurodegenerative disease may actually be a biological defence mechanism against infection.^106,107^ Antimicrobial activity is thought to arise from the ability to rapidly form oligomers and then protofibrils which enable poration of the bacterial cell wall and resultant death. Additionally, these self-assembling structures are also thought to provide a physical barrier to infection *via* entrapment of bacterial cells within β-amyloid fibrillar arrays (Fig. 4).^108^

Several sequences have been shown to exhibit dual complementary amyloid and antimicrobial properties suggesting a functional link between the two.^99^ For example, the human antimicrobial peptide, protegrin-1, which exhibits antimicrobial activity *via* a channel-forming mechanism, also displays the ability to form extended amyloid fibrils analogous to those of classic disease-forming amyloids.^93^ Conversely, Pasupuleti *et al.* examined the antimicrobial activity of full-length amyloid PrP_23–231_ and found that it displayed activity against various Gram-positive and Gram-negative organisms with an exploration of several N- and C-terminally truncated variants indicating that the antimicrobial activity is mediated by the unstructured N-terminus.^109^

The proposal of a functional link between antimicrobial and amyloid peptides has led to the investigation of synthetic amyloid-like peptides which incorporate the FF assembly motif in a bid to develop novel antimicrobials. Rational design often follows one of three distinct pathways:

(1) *de novo* design of supramolecular assemblies,

(2) tuning of properties by remodelling of amyloid-forming sequences to confer the desired functionality,

(3) engineering hybrid complexes consisting of an amyloid core with the addition of non-amyloid sequences to endow the additional desired properties.^96^

Shen and co-workers followed the first pathway and developed the cationic octapeptide KRRFFRRK which presented as a monomer in neutral solutions but self-assembled into amyloid-like fibers in anion solutions (pH > 9.4) and demonstrated permeabilization of the inner and outer membranes of *E*. *coli* resulting in continuous membrane leakage.^110^ Interestingly, the group reported little activity against *S*. *aureus* and postulated that this was due to the thick peptidoglycan coat of Gram-positive organisms insulating against the lytic effects of the peptide. Xu *et al.* followed the second pathway and developed peptide sequences based on previously designed multidomain peptides with the general formula of K_*x*_(QL)_*y*_K_*z*_, which are cationic as well as amphiphilic.^48^ Sequences were found to form fibrils with potent antibacterial activity and minimal cytotoxicity. The optimized sequence, WK_3_(QL)_6_K_2_, composed of both l- and d-form amino acids was demonstrated to display broad spectrum antimicrobial activity against clinically relevant pathogens in the micromolar concentration range. Hu and colleagues developed reversible, self-assembling hydrogelators based on polyphenol-binding amyloid fibrils which displayed antibacterial activity.^111^ The research focused on engineering a hybrid complex in which polyphenols interact with mature amyloid fibrils in high protein concentration to form complex hierarchical structures. The polyphenol-binding amyloid fibrils displayed effective broad spectrum antimicrobial activity by initiating membrane disintegration and agglomeration of both Gram-negative and Gram-positive bacteria. Importantly, the fibrils displayed no significant toxicity against human colonic epithelial cells and the group have suggested potential for biomedical applications such as targeting drug-resistant virulent bacteria and diseases related to infection of the small intestine. Spitzer *et al.* investigated the correlation between amyloid-β peptides of varying length and antimicrobial activity.^112^ Amyloidogenic peptides were found to display broad spectrum antimicrobial activity *via* membrane binding and subsequent cell death with sequences unable to form amyloid fibers displaying little antimicrobial activity. They therefore highlighted a link between propensity to form amyloid fibers and antimicrobial activity and suggested that the secretion of amyloidogenic peptides is part of the innate immune defence within the central nervous system.^113^

Despite several promising results highlighting a correlation between amyloid formation and antimicrobial effect there have also been reports of exceptions to this.^99^ For example, work by Garvey investigating the potential for amyloid formation in antimicrobial and antifungal peptides derived from plant defensins found the antifungal nineteen amino acid fragment RsAFP-19 to be highly amyloidogenic but that fibril formation was independent of and actually inhibited any antifungal activity.^114^ Similarly, Wadhwani and colleagues investigated the influence of stereochemistry in a model amyloidogenic peptide [KIGAKI]_3_-NH_2_ to uncover whether aggregation potential was implicated in the antimicrobial, hemolytic and membrane fusion activities of the molecule.^115^ Functional assays investigating the effects of creating sterically constrained sequences and various substitutions of amino acid enantiomers revealed that the aggregation propensity of the sequence did not influence antimicrobial action. However, this study showed an increased tendency to aggregate promoted other undesirable effects such as hemolysis and membrane fusion.^115^

The research interest in a potential link between amyloid peptides, antimicrobial peptides and the formation of amyloid plaques to date has been reviewed and whilst limited presents an interesting new angle in the use of peptides for antimicrobial applications. Future research will be required to fully elucidate the exact constituents of amyloid peptides required to confer antimicrobial activity whilst further in-depth analysis of the clinical effects and molecular mechanisms behind amyloid plaque formation will be required. Indeed, the establishment of a direct link between amyloid peptides and antimicrobial peptides could result in a paradigm shift in the treatment of neurodegenerative diseases and the development of novel antimicrobial hydrogelators with inherent neuroprotective effects.

## 5. Novel methods for gel characterization

**Fig. 5:**
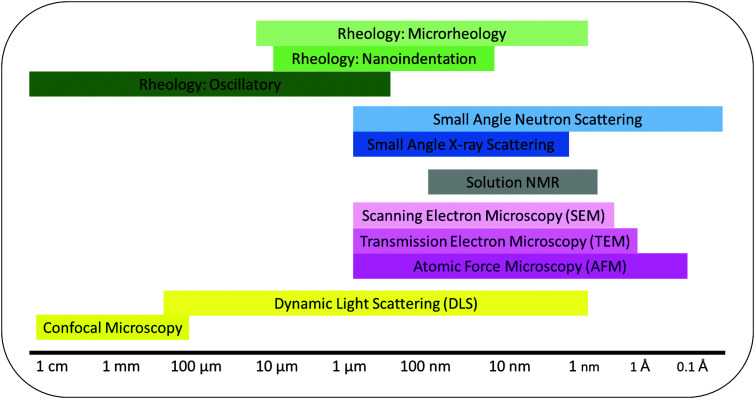
Characterization techniques used to measure self-assembly and gel properties and their functional length scales.

To design hydrogels with the desired structural and antimicrobial properties, determination and characterization of the gel network are of great significance. The common techniques discussed below were chosen from a larger body of methods that provide an insight into the structure of gels. The methods reveal information on the molecular assembly, secondary structures and bulk properties of the gels over several length scales (Fig. 5). Ideal methods to analyze gels must not distort the physical properties of the material nor the chemical interactions between gel components.^116^ To ensure these properties and interactions are not distorted the techniques must have the following characteristics:

• Represent the complete three-dimensional structure and not only the surface.

• Not modify any physical properties of the gel's morphology during the preparation of measurement *e.g.* cryogenically freezing, swelling, pressure changes or placing in a salt buffered solution, avoid methods where samples are manually cut after freezing, coated in gold, or the addition of probe particles.

Where these rules are not followed and the material is distorted before analysis it can result in misleading or invalid data. For example, Mears *et al.* showed how the drying of gels can affect a gel's structural network. Comparing the fiber width of a low molecular weight gel using scanning electron microscopy (SEM), cryo-transmission electron microscopy (cryo-TEM) and small angle neutron scattering (SANS) revealed how the fiber widths differed between gels that were hydrated and dried.^117^ By conducting SEM or cryo-TEM the gel may experience collapsing of the matrix due to vacuum application or the expansion of the matrix due to water freezing. These processes can dramatically modify the gel's morphology making it difficult to make accurate quantitative analysis of the gels without an unknown degree of error.^117^ However, SEM imaging can be very useful to qualitatively analyze different structural regions within a gel and to identify the type of structure formed *e.g.* fibers, spheres, aggregates.^118^

Biophysical characterization of peptide–membrane interactions can be achieved by using structural, calorimetric, spectroscopic, and imaging methods. In this section we have highlighted three techniques we believe are essential for characterizing hydrogels and how they can be used to investigate peptide–membrane interactions.

### 5.1. Light scattering

Light scattering using neutron or X-ray sources *e.g.* small angle neutron scattering (SANS) and small angle X-ray scattering (SAXS) are popular methods to identify physical characteristics of hydrogel structures over several length scales.^116,119^ Both methods are complimentary to each other, however, SANS experiments require the sample to be in a deuterated solvent to avoid unwanted scattering whereas SAXS can be performed in non-deuterated solvents.^116^ Changing a solvent from non-deuterated to deuterated *e.g.* H_2_O to D_2_O can effect self-assembly.^120^ For multidomain hydrogels contrast matching, using a ratio of H_2_O to D_2_O, can be used to identify individual components within the hydrogel network.^121^ It may be possible to increase our understanding of the link between structural characteristics of peptide hydrogelators at the sub-nano scale with microbial cell interactions and antimicrobial efficacy using such techniques.

Neutron scattering can be used to detect water columns inside pores and across membranes. As the peptide interacts with the membrane it experiences morphological change. Grazing-angle neutron diffraction can be used to map the water between the structures during this morphological change.^122^ The use of neutron scattering contrast provided by hydrogel and deuterium labelling can be extremely helpful in locating molecules/functional groups of particular interest such as residues within the peptide. This would allow for information to be elucidated on the specific binding site of the peptide to a membrane.

Dynamic light scattering (DLS) is well known to measure particle hydrodynamic diameter and size distribution of molecules or supramolecular aggregates.^123^ It is also used to determine whether promotion of membrane aggregation in the presence of peptides in solution occurs. DLS has been used to determine membrane surface charge (*ζ*-potential) promoted by peptide–membrane interactions. The data obtained by these techniques yields valuable descriptions of peptide–membrane interactions.^124^ The peptide–membrane interactions often lead to changes in the physical properties of the membrane such as size, charge, and shape. Membrane-active peptides vary in their hydrophobic and charged amino acid contents. These peptide properties are believed to determine their interaction-related effects on membranes.^125^

Many biological systems like bacterial membranes are composed of negatively charged lipids which result in a negative charge on the surface. In particular, Gram-negative bacteria which also contain endotoxins and lipopolysaccharides have a highly negatively charged surface. Using DLS the *ζ*-potential of the membrane surface can be measured. When an antibiotic or antimicrobial peptide binds to the surface of the membrane the overall surface charge should decrease. Therefore, the degree to which a peptide can bind to a membrane surface can be analyzed and a prediction of bactericidal efficacy can be deduced.

### 5.2. Rheology

Rheology is a common tool used to measure the macroscopic properties of hydrogels including homogeneity, strength and stiffness.^116,126^ These are becoming increasingly important characteristics in the design and understanding of anti-infective peptide gelators. The most frequently used instrument in literature is the oscillatory rheometer and this technique measures mechanical properties of bulk gels with sample size of at least 0.5 mL.^126^ Other rheological methods including nanoindentation which uses an AFM microscope and cantilever to measure the rheology of individual fibers, are less common. Nanoindentation can be useful for measuring different regions within a gel to identify homogeneity, or multiple domains.^127^ It is worth noting that the elastic and loss moduli of individual fibers do not necessarily represent the entire network or its bulk rheology. Another rheological method, microrheology, uses dynamic light scattering to probe tracer particles which identify the elastic and liquid moduli. Microrheology requires the addition of a probe, usually polystyrene nanoparticles, which when added before gelation can distort the self-assembly of the hydrogels. However, this method is an example of *in situ* rheology.^128^ Finally, cavitational rheology which exploits the relationship between the internal and external pressures when an air bubble injected by a needle of specified diameter is burst.^129^ This relationship yields the elastic and liquid moduli. Cavitational rheology provides *in situ* rheology and can identify differences in rheology between different areas and depths of the sample.

### 5.3. Nuclear magnetic resonance (NMR)

Self-assembly kinetics, fiber-fiber interactions and pH changes can be measured *in situ* using NMR. Recent advances by Wallace *et al.* have shown how NMR spectroscopy can be used to determine the gelator p*K*_*a*_ and pore size during gelation.^130–132^ The self-assembly process can be followed by NMR detectable gelator molecules *e.g.* <25 kDa. Large gelator structures experience slow tumbling in solution which leads to faster relaxation of transverse magnetization, this causes the gelator to appear invisible in the spectra.^133^ The NMR peaks are then integrated against a known standard to determine gelator concentration over time.^134^ This method can be of particular importance for multidomain hydrogel self-assembly.^121,134^ By following each individual component of the multidomain mixture the self-assembly can be followed and possibly linked to antimicrobial mechanism of action. If one component self-assembles before the second component begins to assemble then this can be used to identify a self-sorted system. Parameters that control the rate of gelation can be adjusted and the corresponding change in individual component self-assembly measured. By changing the rate of individual component self-assembly properties such as entanglement of fibers, gel stiffness/strength and color can be controlled.^121^

## 6. Future perspectives

Linking the fundamental structure and architecture of peptide hydrogel systems to antimicrobial mechanism of action and selectivity will have important implications for future developments in the wider fields of soft matter and material science. Currently the need for new anti-infective treatments appears greatest, with increasing demands on existing antimicrobial formularies and rising resistance to these treatments observed clinically.^135^ Peptide hydrogels have the potential to play a key role in resolving this crisis due to their tunability, enabling precise modification of chemical and functional properties that should have a direct impact on important characteristics.^136^ This includes the mechanical properties of the gel formulation which can be optimized to improve its ease of administration; ability to recover from shear stress or reside within a wound cavity and most interestingly its underlying antimicrobial spectrum of activity (selectivity for specific bacteria and/or reduced toxicity against host cells and tissues).^137,138^ Peptide hydrogels may also be combined with existing antimicrobial drugs, acting synergistically to reduce their clinically effective concentrations, thereby improving efficacy of combined treatments and reducing the overall risk of generating antimicrobial resistance.^88^

Most healthcare professionals prefer a clinical scenario whereby antimicrobials are prescribed based on efficacy of a drug against a very specific diagnosed causative pathogen(s), rather than a broad spectrum empirical approach to therapy.^139^ This is in order to reduce the impact of therapy on the host microbiota, whilst simultaneously lowering the potential for treatment failure and drug resistance. In future, peptide hydrogel structure and mechanical strength may be implicitly linked to antimicrobial action and they may therefore be shown to exert their effect primarily by physical means.^55^ For example, by acting like a fibrous antimicrobial sponge to selectivity compromise microbial membranes and intracellular contents. This may be perceived as a more promising mode of action when compared to existing drug treatment strategies which target individual biomolecular pathways. A technology which targets bacteria by physical means would reduce the potential for and long-term impact of antimicrobial resistance development. If peptide hydrogels could be further optimized to target individual species or strains of infective microorganisms then this would bring huge clinical benefit.

The scope for changeable mechanical properties ensures peptide hydrogels are amenable to a variety of microbiology applications. For example, their soft gel nature and syringe-ability, combined with potential for shear recovery, means improved ease of administration to patients *via* injection in order to treat localized infection sites.^140^ This strategy would likely yield decreased systemic side effects and reduced exposure to commensal microorganisms, reducing resistance development and impact on host microbiota. The most obvious use for such a gel formulation would be as a wound-healing product, with a peptide-based hydrogel providing a biocompatible environment similar to host tissue in order to support skin cell growth, primarily *via* keratinocyte and dermal fibroblast migration and angiogenesis.^141^ Wounds, by their very nature are complex, and in their most simple context are defined as acute or chronic. Chronic wounds, for example diabetic wounds, remain unresolved after weeks due to an underlying inability for the patient's body to heal.^142^ Chronic wounds are often associated with an aggravated host inflammatory response which increases cell and tissue damage at the wound site. Once infected the clinical picture becomes further complicated. Initial stages of bacterial infection are predominantly Gram-positive in nature (*e.g. S. aureus*), whereas Gram-negative infection (*e.g. P. aeruginosa*, *E. coli*) dominate chronic infected wounds.^143^ Further research aimed at tailoring antimicrobial peptide hydrogels to be species specific would enable a treatment to be utilized for each stage of infection. The potential for a personalised approach to treatment using such systems and tailored to each individual patient becomes even more encouraging when combined with innovative methods of manufacture. For example, in future 3D printing may allow a peptide hydrogel dressing to be manufactured that fits to the shape of the wound cavity.^144^ Additionally, a layer-by-layer approach may allow separate hydrogel systems to be included within one wound healing product. There is the potential to have an initial base layer that acts to treat acute wounds and/or primarily Gram-positive based infection. Once this layer is absorbed or dissolved a separate layer may provide anti-inflammatory activity alongside targeted selectivity for Gram-negative pathogens and/or conditions to promote cell growth and healing. A peptide hydrogel would also provide the means to enhance the co-delivery of stem cells to areas where infection may also be present or is required to be prevented. For example, the delivery of pluripotent stem cells after dental root canal extraction or for a long-acting injectable implant where the introduction of infection is a risk and may wish to be prevented (ocular and spinal implants).^145–147^

The potential for the peptide sequence to be modified in order to respond to infection or infectious stimuli (*e.g.* specific enzymes or pH change) is of major importance to the development of future technologies in this area. As highlighted within our introduction, a peptide hydrogel coating that could provide long-term protection against infection for medical devices, especially implants such as urinary catheters or more permanent devices such as heart valves and hip replacements would be of significant societal benefit. There is expected to be an increased reliance on biomaterials in future, coinciding with an increased life expectancy and older population demographic.^138^

Whilst peptide hydrogels may prove useful as future antimicrobial treatments, as highlighted by the examples throughout this review, they may also serve as a potential diagnostic platforms for identifying infectious disease. Hydrogel formation provides an important role in confirming the presence of lipopolysaccharides derived from Gram-negative bacterial endotoxins. The hydrogel forming enzyme limulus amebocyte lysate, derived from horseshoe crab, responds to the presence of endotoxin and in this way is used as an assay in the manufacture of sterile pharmaceutical formations *e.g.* injectables, eye drops.^148^ This is important as the presence of endotoxins *in vivo* can elicit a severe host immune response. If peptide hydrogel formation could similarly be tailored to respond to specific stimuli, a rapid diagnostic test for particular microorganisms, especially drug resistant strains, could be developed and therefore more effective treatment regimens could be selected accordingly.^149–151^

One future benefit to microbiology of soft matter and hydrogel research, which often is not considered, is the use of inert peptide hydrogels as a platform to study the development of infectious disease, most notably the role of microbial biofilms. This would mirror a similar approach to the use of hydrogels in 3D cell culture and the progress of tumour organoids in cancer research only in a microbial cell setting.^152^ Peptide hydrogels could be tailored to serve as gel platforms to study the growth and maturation of planktonic bacteria into the biofilm phenotype. Capturing the early stage transition from planktonic to biofilm form would be especially beneficial. Biofilms are primarily composed of cells embedded within an extracellular polysaccharide gel matrix. Whilst they are primarily composed of polysaccharide sugars, there is ability to introduce sugars to the peptide chain in order to more closely replicate the biofilm environment. Carbohydrate and dual carbohydrate-peptide based gelators also exist and could be utilized in such an approach.^153^ There are similarities between peptide hydrogel and biofilms with regards to their soft gel composition. The fibrous nature of biofilms and their increasing tolerance to several licensed drug treatments, ultimately by reducing their diffusion and exposure to bacterial cells, is often replicated by peptide hydrogels for the sustained release of drugs. A key question would be how crucial is hydrogel stiffness to playing a role in controlling bacterial cell function and cell fate? Similar questions have been posed in the differentiation of human mesenchymal stem cells using tissue-like hydrogel matrices.

Precise exploration of biofilm formation, survival and growth at a biomolecular and genetic level using peptide hydrogels would enable the development of new drug targets aimed at preventing biofilm formation. If bacteria could remain in the planktonic liquid phenotype they would likely prove to be more susceptible to existing antimicrobial strategies and the risk of resistance would be lessened given that biofilm tolerance is reduced and therefore clinically relevant concentrations of antimicrobial therapies are more likely to be achieved at the infection site.^154^ Peptide hydrogels would also provide a platform for studying biofilm and microbial response to existing and future treatments. For example, they could be utilized to study how bacterial stress response pathways including the upregulation of *marA*/soxS antibiotic-resistance and superoxide stress response regulators and the *baeSR, cpxAR, kdpDE, phoQP, rcsCB* two-component systems, respond to drugs of interest.^59,155,156^ Mammalian cells and tissues could be incorporated alongside microbial cells to gain a truer reflection of the infection environment present within the host. Ultimately such infectious disease models may be used to generate data relating to safety and efficacy, in future serving as an *ex vivo* alternative to animal models. Similar models could be designed to study the possible role of infection and protein plaques in Alzheimer's disease and be used to identify novel treatment strategies, an area which has been severely limited in recent years, despite the recent controversial approval of aducanumab by the FDA.^157^

Peptide hydrogels are now widely explored as solutions to bacterial and less commonly fungal-based infections. However, future study may also explore their role as tailored therapies to target viral infection. Self-assembled peptides have been studied as vaccine adjuvants, demonstrating promise as alternatives to aluminium-based excipients, but their use as treatment strategies is less studied outside of research into the potential role of antimicrobial peptides in treating HIV/AIDS.^158^ Agriculture also offers a wealth of opportunities for a designed anti-infective peptide hydrogel platform. Such a technology could be utilized as a treatment for bovine mastitis in cows or as alternative to soils or seed coatings in agriculture.^159^ The peptide could be tailored to prevent fungal or bacterial contamination of food products, alongside an approved safety profile as required by regulators. This could be a more environmentally friendly solution compared to existing herbicidal and pesticidal products.

Whilst each of these approaches are promising for the development of future technologies, key issues still have to be addressed to clarify the impact of peptide hydrogels to microbiology. Most notably:

• What are the chemical and structural components that are essential for selective microbicidal activity in these systems, both between mammalian *vs.* microbial cells and across different microbial strains (*e.g.* Gram-positive *vs.* Gram-negative) or isolates (*e.g.* MRSA *vs.* drug susceptible *S*. *aureus*)? What is the reason for such selectivity?

• Is there a minimum peptide hydrogel forming motif that can be generated to provide all the chemical and structural components required for selective activity?

• What is the influence on antimicrobial activity and self-assembly of the quantity and structural position of amino acids and specific chemical functional groups within the peptide hydrogel motif?

• What is the exact mechanism and target(s) of inhibitory or microbicidal activity in peptide hydrogels and the corresponding inhibition/kill kinetics? Does this significantly differ in gel and soluble forms?

• How can peptide hydrogels be best integrated with existing medical approaches employed to alleviate or prevent infection *e.g.* existing licensed antimicrobials, medical device coatings?

## 7. Conclusions

This review demonstrates the promise of peptide hydrogels as future antimicrobial platforms. A major benefit of this approach is the inherent antimicrobial activity provided by the hydrogel forming peptide molecule. Peptides also provide the chemical versatility and choice of chemical functional groups to enabling tailoring of functional properties (*e.g.* mechanical strength, spectrum of antimicrobial activity) to desired purpose. Significant questions remain as to how these systems fundamentally interact with microorganisms to achieve selective antimicrobial efficacy. This will form an important area of future research, enabling the design of peptide-based platforms for use as innovative anti-infective and infection responsive materials within the areas of medical devices, wound healing, drug delivery and 3D cell culture. Additional considerations for future clinical translation to patients include overcoming the challenges faced by antimicrobial peptides over the past three decades. These include demonstrating: reduced toxicity upon local and systemic administration; combined pharmaceutical and biological stability; cost-effective and efficient upscale in pharmaceutical manufacture; and an ability to be delivered at clinically effective concentrations locally to the site of infection with reduced propensity for the development of antimicrobial resistance. These challenges can be addressed through purposeful collaboration between multidisciplinary scientists (microbiologists, peptide chemists, physicists), industry and the pharmaceutical regulators.

## Author contributions

The manuscript was written through contributions of all authors. All authors have given approval to the final version of the manuscript. ERC, SMC, SP and GL were involved in writing the original draft, reviewing and editing. GL conceptualized the manuscript.

## Conflicts of interest

There are no conflicts to declare.

## Supplementary Material
